# Prefrontal theta modulates sensorimotor gamma networks during the reorienting of attention

**DOI:** 10.1002/hbm.24819

**Published:** 2019-10-17

**Authors:** Rachel K. Spooner, Alex I. Wiesman, Amy L. Proskovec, Elizabeth Heinrichs‐Graham, Tony W. Wilson

**Affiliations:** ^1^ Department of Neurological Sciences University of Nebraska Medical Center (UNMC) Omaha Nebraska; ^2^ Center for Magnetoencephalography University of Nebraska Medical Center Omaha Nebraska; ^3^ Department of Psychology University of Nebraska Omaha Nebraska

**Keywords:** magnetoencephalography, oscillations, Posner cueing task, top‐down

## Abstract

The ability to execute a motor plan involves spatiotemporally precise oscillatory activity in primary motor (M1) regions, in concert with recruitment of “higher order” attentional mechanisms for orienting toward current task goals. While current evidence implicates gamma oscillatory activity in M1 as central to the execution of a movement, far less is known about top‐down attentional modulation of this response. Herein, we utilized magnetoencephalography (MEG) during a Posner attention‐reorienting task to investigate top‐down modulation of M1 gamma responses by frontal attention networks in 63 healthy adult participants. MEG data were evaluated in the time–frequency domain and significant oscillatory responses were imaged using a beamformer. Robust increases in theta activity were found in bilateral inferior frontal gyri (IFG), with significantly stronger responses evident in trials that required attentional reorienting relative to those that did not. Additionally, strong gamma oscillations (60–80 Hz) were detected in M1 during movement execution, with similar responses elicited irrespective of attentional reorienting. Whole‐brain voxel‐wise correlations between validity difference scores (i.e., attention reorienting trials—nonreorienting trials) in frontal theta activity and movement‐locked gamma oscillations revealed a robust relationship in the contralateral sensorimotor cortex, supplementary motor area, and right cerebellum, suggesting modulation of these sensorimotor network gamma responses by attentional reorienting. Importantly, the validity difference effect in this distributed motor network was predictive of overall motor function measured outside the scanner and further, based on a mediation analysis this relationship was fully mediated by the reallocation response in the right IFG. These data are the first to characterize the top‐down modulation of movement‐related gamma responses during attentional reorienting and movement execution.

## 1 INTRODUCTION

Our ability to select and execute a movement in response to environmental and cognitive demands generally requires higher order attentional allocation to behaviorally relevant stimuli. Such allocation (and reallocation) of attentional resources to pertinent information has been classically studied using the Posner cueing task (Posner, 1980). During this task, participants are generally given a cue that precedes a target stimulus, the location of which can either be the same (validly cued) or different (invalidly cued) as the location of the target. This gives rise to a behavioral phenomenon termed the validity effect (Vossel, Thiel, & Fink, 2006), whereby participants are slower in responding to invalidly cued targets due to the cost of reorienting attention (Corbetta, Patel, & Shulman, 2008). While previous studies have identified regions in the dorsal and ventral attention networks serving attentional reorienting (Corbetta et al., 2008; Doricchi, Macci, Silvetti, & Macaluso, 2010; Indovina & Macaluso, 2007; Leitão, Thielscher, Tünnerhoff, & Noppeney, 2015; Macaluso & Patria, 2007; Petersen & Posner, 2012; Proskovec, Heinrichs‐Graham, Wiesman, McDermott, & Wilson, 2018; Shulman & Corbetta, 2012; Thiel, Zilles, & Fink, 2004; Vossel, Geng, & Fink, 2014; Xuan et al., 2016), the potential for top‐down influence of such higher order regions on oscillatory activity in primary sensory and motor regions has not been widely investigated.

Motor control is known to be served by two specific patterns of beta (15–30 Hz) oscillatory activity, including the perimovement beta event‐related desynchronization (ERD) and the postmovement beta rebound (PMBR). These responses have been linked to distinct phases of motor control; the perimovement ERD has been associated with the planning and execution of movements and is known to be modulated by many planning‐related cognitive factors (Grent‐'t‐Jong, Oostenveld, Jensen, Medendorp, & Praamstra, 2014; Heinrichs‐Graham, Arpin, & Wilson, 2016; Heinrichs‐Graham & Wilson, 2015; Tzagarakis, Ince, Leuthold, & Pellizzer, 2010), while the PMBR is thought to be critical to the termination of movements (Heinrichs‐Graham, Kurz, Gehringer, & Wilson, 2017; Jurkiewicz, Gaetz, Bostan, & Cheyne, 2006; Pfurtscheller & Lopes da Silva, 1999). There is also a third, more transient oscillatory response in the gamma range (>30 Hz) that coincides with movement onset and is transient (Cheyne, Bells, Ferrari, Gaetz, & Bostan, 2008; Gaetz, Edgar, Wang, & Roberts, 2011; Gaetz, Macdonald, Cheyne, & Snead, 2010; Muthukumaraswamy, 2010; Wilson et al., 2010). This so‐called movement‐related gamma synchrony (MRGS) is thought to reflect a motor execution signal in the primary motor (M1) cortices, and has been shown to be modulated by multiple movement parameters, such as the type of movement and muscle groups involved (Muthukumaraswamy, 2010). However, recent evidence has additionally connected this response to higher order processing. For example, Gaetz et al. used a multisource response interference task to probe the functionality of the MRGS and found that responses in the contralateral M1 occurred earlier during interference relative to control trials (Gaetz, Liu, Zhu, Bloy, & Roberts, 2013). Similarly, Grent‐'t‐Jong et al. found an earlier MRGS during times of response conflict relative to no conflict using a modified Eriksen flanker task (Grent‐'t‐Jong, Oostenveld, Jensen, Medendorp, & Praamstra, 2013). Beyond these latency findings, a more recent study reported spectrally specific conditional modulation of the MRGS during a flanker task, such that the peak gamma frequency was significantly higher in trials with incongruent relative to congruent distractors (Heinrichs‐Graham, Hoburg, & Wilson, 2018). Together, these findings suggest that the MRGS response may indeed be sensitive to higher order task parameters.

While extensive evidence links beta and gamma oscillations in the motor cortex to the planning, selection, and execution of voluntary movements, the degree to which these responses are modulated by higher order attentional processes is not fully understood. The ability to orient and reorient attention to relevant sensory information and react accordingly is essential to behavior. Thus, the goal of the current study was to identify the impact of higher order cognitive control on bottom‐up motor responses in the context of attentional reorienting. To this end, we utilized magnetoencephalography (MEG) during performance of a Posner cueing task in a large sample of healthy adults and hypothesized that prefrontal oscillatory activity associated with attentional reorienting would uniquely modulate the MRGS response during task performance, and that such gamma activity would be related to motor functioning.

## 2 METHODS AND MATERIALS

### 2.1 Participants

We studied 63 healthy adults (32 males; mean age: 36.06, range: 22–55, 52 right‐handed) who were recruited from the local community. Exclusionary criteria included any medical illness affecting CNS function, neurological disorder, history of head trauma, current substance abuse, and the MEG Laboratory's standard exclusion criteria (e.g., dental braces, metal implants, and/or any type of ferromagnetic implanted material). After a complete description of the study, written informed consent was obtained from participants following the guidelines of the University of Nebraska Medical Center's Institutional Review Board, which approved the study protocol.

### 2.2 Experimental paradigm

**Figure 1:**
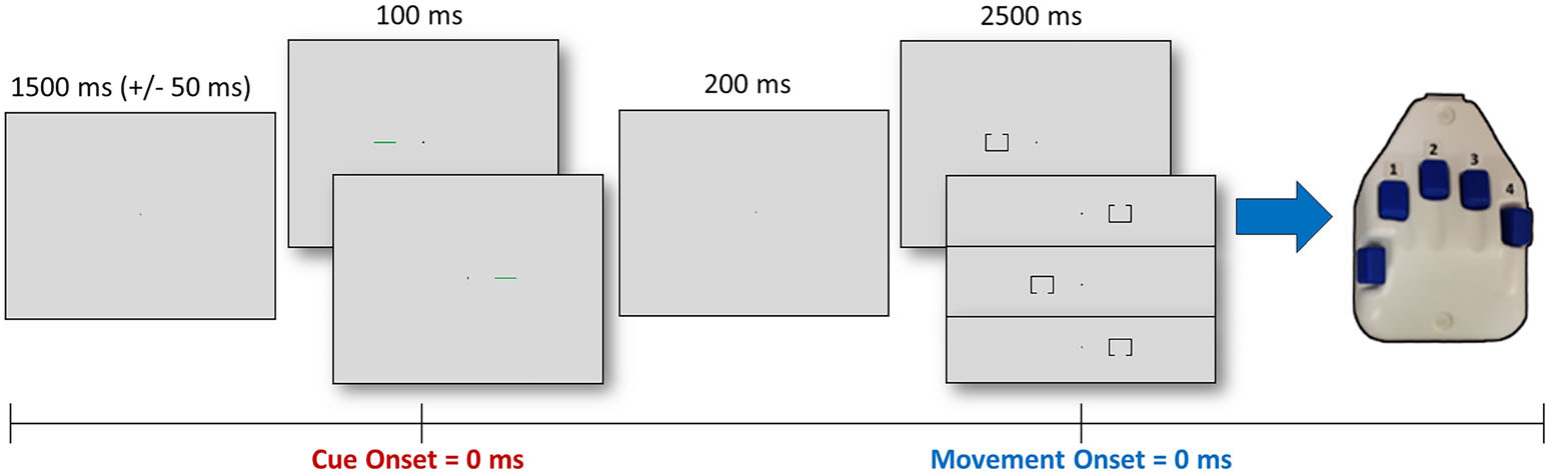
Posner cueing task and epoch definition. A fixation cross was presented for 1,500 (±50) ms, followed by a cue (green bar) presented in the left or right visual hemifield for 100 ms. After 200 ms, the target stimulus (box with opening) appeared in either the left or right visual hemifield for 2,500 ms. Participants responded as to whether the opening was on the bottom or top of the target with their index and middle fingers, respectively. The cue was valid (presented on the same side as the subsequent target) 50% of the time. To evaluate the responses involved in attentional reorientation (cue‐locked), the neuromagnetic data were defined with the onset of the cue as 0 ms (denoted in red) and the baseline was defined as the 600 ms preceding cue onset. To evaluate motor responses (movement‐locked), the data were defined with movement onset as 0 ms (denoted in blue) and the baseline was defined as a 600 ms period prior to movement (and prior to the cue onset) that was individually adjusted based on reaction time [Color figure can be viewed at http://wileyonlinelibrary.com]

During MEG recording, participants sat in a nonmagnetic chair within a magnetically shielded room and performed a modified Posner task (Figure 1; Posner, 1980). Participants were instructed to maintain fixation on a centrally presented crosshair throughout the task. Each trial began with the presentation of only the crosshair for 1,500 ms (±50 ms). Next, a green bar, serving as the cue, was presented either to the left or right of the crosshair for 100 ms. This cue was presented on each side (left or right) an equal number of times, and could be either valid (i.e., presented on the same side as the subsequent target; 50% of all trials) or invalid (i.e., opposite side relative to the target). After 100 ms, the cue disappeared and 200 ms later (i.e., 300 ms after cue onset) the target stimulus appeared on either the left or right side of the crosshair for 2,500 ms. The target consisted of a box with an opening on either the bottom (50% of trials) or top surface. Participants were instructed to respond as to whether the opening was on the bottom (right index finger) or the top (right middle finger) of the box. Each target variant appeared an equal number of times on the left and right sides of the crosshair, and was preceded by an invalid or valid cue an equal number of times. Each trial lasted 4,300 ms (±50 ms) and there were a total of 200 trials (100 valid, 100 invalid), resulting in a total run time of approximately 14.5 min. Additionally, participants completed the Grooved Pegboard task with dominant and nondominant hands and a composite score was generated to assess overall motor function outside of the scanner.

### 2.3 MEG data acquisition

Recordings were conducted in a one‐layer magnetically shielded room with active shielding engaged. With an acquisition bandwidth of 0.1–330 Hz, neuromagnetic responses were sampled continuously at 1 kHz using an Elekta MEG system with 306 magnetic sensors (Elekta, Helsinki, Finland). MEG data from each participant were individually corrected for head motion and subjected to noise reduction using the signal space separation method with a temporal extension (Taulu & Simola, 2006; Taulu, Simola, & Kajola, 2005).

### 2.4 Structural MRI acquisition, processing, and coregistration with MEG data

Preceding MEG measurement, four coils were attached to the subject's head and localized, together with the three fiducial points and scalp surface, with a 3D digitizer (Fastrak 3SF0002; Polhemus Navigator Sciences, Colchester, VT). Once the subject was positioned for MEG recording, an electric current with a unique frequency label (e.g., 322 Hz) was fed to each of the coils. This induced a measurable magnetic field and allowed each coil to be localized in reference to the sensors throughout the recording session. Since coil locations were also known in head coordinates, all MEG measurements could be transformed into a common coordinate system. With this coordinate system, each participant's MEG data were coregistered with their structural T1‐weighted neuroanatomical data prior to source space analyses using BESA MRI (Version 2.0; BESA GmbH, Gräfelfing, Germany). These data were acquired with a Philips Achieva 3T X‐series scanner using an eight‐channel head coil (TR: 8.09 ms; TE: 3.7 ms; field of view: 240 mm; slice thickness: 1 mm; no gap; in‐plane resolution: 1.0 × 1.0 mm^2^). All structural MRI data were aligned parallel to the anterior and posterior commissures and transformed into standardized space, along with the functional images, after beamforming.

### 2.5 MEG Time–Frequency Transformation and Statistics

Cardiac and ocular artifacts (e.g., blinks, eye movement) were removed from the data using signal‐space projection (SSP), which was accounted for during source reconstruction (Uusitalo & Ilmoniemi, 1997). MEG data were then analyzed with respect to the attentional cue (cue‐locked) and response onset (movement‐locked) individually to evaluate the oscillatory dynamics associated with attentional reorientation and movement selection, respectively. To evaluate the higher order responses involved in attentional reorientation, the continuous magnetic time series was divided into epochs of 4,000 ms duration, with the onset of the cue being defined as 0 ms (cue‐locked) and the baseline being defined as the 600 ms preceding cue onset (i.e., −600 to 0 ms). Given our task and epoch design, the target onset occurred at 300 ms. Conversely, to evaluate primary motor responses, epochs were defined with movement onset as 0 ms (movement‐locked) and the baseline being a 600 ms period preceding movement onset that was also prior to cue presentation. Essentially, the exact location of the 600 ms window was adjusted based on the participant's reaction time (RT) to ensure the cue and target visual stimuli were not presented during the baseline. Note that these baseline periods were selected to prevent the PMBR (in the cue‐locked analysis) and the presentation of visual stimuli (movement‐locked analysis) from “contaminating” the baseline. Epochs containing artifacts were rejected based on a fixed threshold method, supplemented with visual inspection. In brief, for each individual, the distribution of amplitude and gradient values was computed across all trials, and those trials containing the highest amplitude and/or gradient values relative to the full distribution were rejected by selecting a threshold that excluded extreme values. Importantly, these thresholds were set individually for each participant, as interindividual differences in variables such as head size and proximity to the sensors strongly affects MEG signal amplitude. Additionally, we visually inspected the data to identify trials contaminated with other types of artifacts, such as those produced by muscle tension, and rejected such trials. On average, 86.3 valid and 86.1 invalid cue‐locked trials per participant remained after artifact rejection, and these were used in subsequent analyses. Similarly, on average 86.3 valid and 85.7 invalid movement‐locked trials remained after artifact rejection. Importantly, the number of trials did not significantly differ based on condition for cue‐ or movement‐locked analyses (*p*'s > .36). Artifact‐free epochs were transformed into the time–frequency domain using complex demodulation with a resolution of 2 Hz and 25 ms, and the resulting spectral power estimations per sensor were averaged across all trials to generate time–frequency plots of mean spectral density. These sensor‐level data were then normalized with respect to baseline power, which was calculated as the mean power during the 600 ms period prior to cue onset for both movement‐locked and cue‐locked analyses. Of note, this normalization was performed separately for each 2 Hz by 25 ms bin within each spectrogram using the corresponding baseline data.

The time–frequency windows used for imaging were determined by statistical analysis of the sensor‐level spectrograms across all trials (valid + invalid), gradiometers, and participants. Each data point (i.e., 2 Hz by 25 ms bin) in the spectrogram was initially evaluated using a mass univariate approach based on the general linear model. To reduce the risk of false positive results while maintaining reasonable sensitivity, a two‐stage procedure was followed to control for Type 1 error. In the first stage, paired‐sample *t* tests were conducted on each data point and the output spectrogram of *t* values was thresholded at *p* < .05 to define time–frequency bins containing potentially significant oscillatory deviations across all participants. In Stage 2, time–frequency bins that survived this threshold were clustered with temporally and/or spectrally neighboring bins that were also significant, and a cluster value was derived by summing all of the *t* values of all data points in the cluster. Nonparametric permutation testing was then used to derive a distribution of cluster values and the significance level of the observed clusters (from stage one) were tested directly using this distribution (Ernst, 2004; Maris & Oostenveld, 2007). For each comparison, at least 10,000 permutations were computed to build a distribution of cluster values. Based on these analyses, only the time–frequency windows that contained significant oscillatory events across all trials were subjected to the beamforming (i.e., imaging) analysis. Thus, a data‐driven approach was utilized for selecting the time–frequency windows to be imaged. Note again that statistical analysis of sensor‐level data was done separately for the cue‐locked and movement‐locked analysis, as the oscillatory responses were expected to be very different between the two analyses and this allowed each subset to be identified using our data‐driven approach.

### 2.6 MEG Source Imaging and Statistics

Cortical networks were imaged through the dynamic imaging of coherent sources beamformer (Gross et al., 2001), which applies spatial filters to time–frequency sensor data to calculate voxel‐wise source power for the entire brain volume. Such images are typically referred to as pseudo‐t maps, with units (pseudo‐t) that reflect noise‐normalized power differences (i.e., active vs. passive) per voxel. Following convention, the source power in these images was normalized per participant using a separately averaged prestimulus noise period (i.e., baseline) of equal duration and bandwidth (Hillebrand, Singh, Holliday, Furlong, & Barnes, 2005). MEG preprocessing and imaging used the Brain Electrical Source Analysis (version 6.0) software.

Normalized source power was computed for the selected time–frequency bands over the entire brain volume per participant at 4.0 × 4.0 × 4.0 mm^3^ resolution. Each participant's functional images were transformed into standardized space using the transform that was previously applied to the structural images and then spatially resampled. The resulting 3D maps of brain activity reflected activity across both conditions (i.e., valid and invalid) and were averaged across participants to assess the anatomical basis of the significant oscillatory responses identified through the sensor‐level analysis. Note that this was done separately for the cue‐locked and movement‐locked maps. To identify the effect of validity on top‐down oscillatory responses, we used the cue‐locked data and performed a whole‐brain analysis of the validity effect (i.e., invalid vs. valid) using paired‐sample *t* tests for the oscillatory responses of interest, as determined by the sensor‐level statistical analyses. To account for multiple comparisons, a significance threshold of at least *p* < .01 was used for the identification of significant clusters in all whole‐brain statistical maps, accompanied with a stringent cluster (*k*) threshold of at least 500 contiguous voxels. From these significant clusters, pseudo‐t values per condition were then extracted from the peak voxels of each significant cluster in the resulting “validity effect” maps.

## 3 RESULTS

### 3.1 Behavioral analysis

Two participants were excluded from all analyses due to their MEG data being severely contaminated with artifacts. The remaining 61 participants performed well, accurately responding to 99.35% (*SD* = 0.96%) of the valid trials and 98.85% (*SD* = 1.59%) of the invalid trials. This accuracy difference was significant *t*(60) = −2.36, *p* = .022. Additionally, there was a significant difference in RT between conditions *t*(60) = 7.89, *p* < .001, with participants responding more slowly during invalid trials (*M* = 822.53 ms, *SD* = 180.04 ms) relative to valid trials (*M* = 767.38 ms, *SD* = 176.96 ms). Thus, the mean validity difference effect was 55.15 ms (*SD* = 5.41), which is consistent with previous work (Proskovec et al., 2018; Vossel et al., 2006).

### 3.2 Sensor‐level analysis

**Figure 2:**
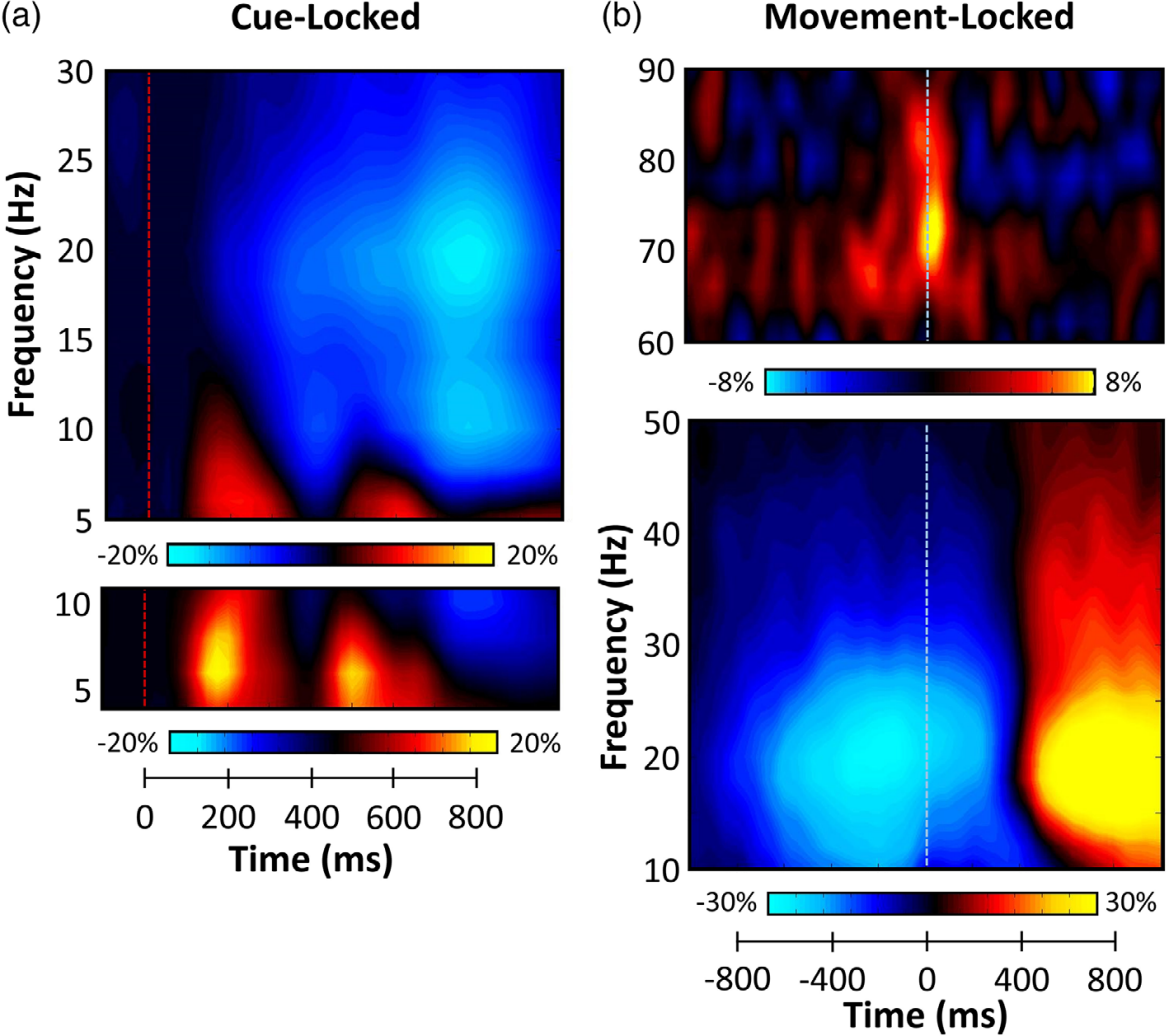
Magnetoencephalography (MEG) sensor‐level spectrograms. (a) Cue‐locked time–frequency spectrograms for two sensors near the parietal cortices. The *x* axis denotes time (ms) with the onset of the cue occurring at 0 ms (red dotted line) and target onset occurring at 300 ms. The *y* axis represents frequency (Hz). Power is shown in percentage units relative to the baseline period (−600 to 0 ms), with color scale bars beneath the spectrograms. Data have been averaged across all trials and participants. Strong decreases in alpha (8–14 Hz) and beta (14–22 Hz) oscillations were observed at the onset of the target stimulus. Additionally, large increases in theta (4–8 Hz) activity were seen following the cue and during target processing. (b) Movement‐locked time–frequency spectrograms for two sensors near the sensorimotor cortices. The *x* axis denotes time (ms) with the onset of movement occurring at 0 ms (blue dotted line) and the *y* axis represents frequency (Hz). Percent power changes relative to the baseline period (600 ms period preceding cue onset) are shown as color scales beneath each spectrogram. Strong decreases in beta (16–26 Hz) activity were observed prior to and after movement onset. Additionally, transient increases in gamma (66–76 Hz) activity were seen during movement onset. Finally, strong increases in beta (16–26 Hz) activity (i.e., the PMBR) were observed following movement termination [Color figure can be viewed at http://wileyonlinelibrary.com]

Statistical analysis of the cue‐locked time–frequency spectrograms revealed significant clusters of theta (4–8 Hz), alpha (8–14 Hz), and beta (14–22 Hz) oscillatory activity in gradiometers near the occipital and parietal cortices across all participants and conditions (*p* < .001, corrected; Figure 2). While strong theta and alpha visual responses were seen shortly after the onset of the cue, we focused our analyses on oscillatory activity during the target interval, as we were mainly interested in the attentional reorienting aspect. Briefly, significant theta activity began about 125 ms after the onset of the target stimulus (300 ms = target onset) and tapered off about 250 ms later (i.e., from 425 to 675 ms). Neural responses in the alpha and beta range were much more extended, with significant activity in both bands emerging shortly after target onset and continuing for about 600 ms before dissipating (i.e., from 300 to 900 ms).

Statistical analysis of the movement‐locked time–frequency spectrograms revealed significant beta (16–26 Hz) ERD responses in gradiometers near the bilateral sensorimotor cortices across all participants and conditions (*p* < .001, corrected; Figure 2), which extended from approximately 500 ms before movement onset until about 300 ms after (0 ms = movement onset). Likewise, a strong PMBR (16–26 Hz) was detected during the 800–1,400 ms time period in roughly the same set of gradiometers near sensorimotor cortices. Finally, significant gamma (66–76 Hz) synchronization was observed in a subset of left sensorimotor gradiometers, which extended from approximately 100 ms prior to movement onset until 100 ms after the movement was initiated. These neural responses correspond closely to the perimovement beta ERD, PMBR, and MRGS responses identified in many previous studies.

### 3.3 Beamformer analysis

**Figure 3:**
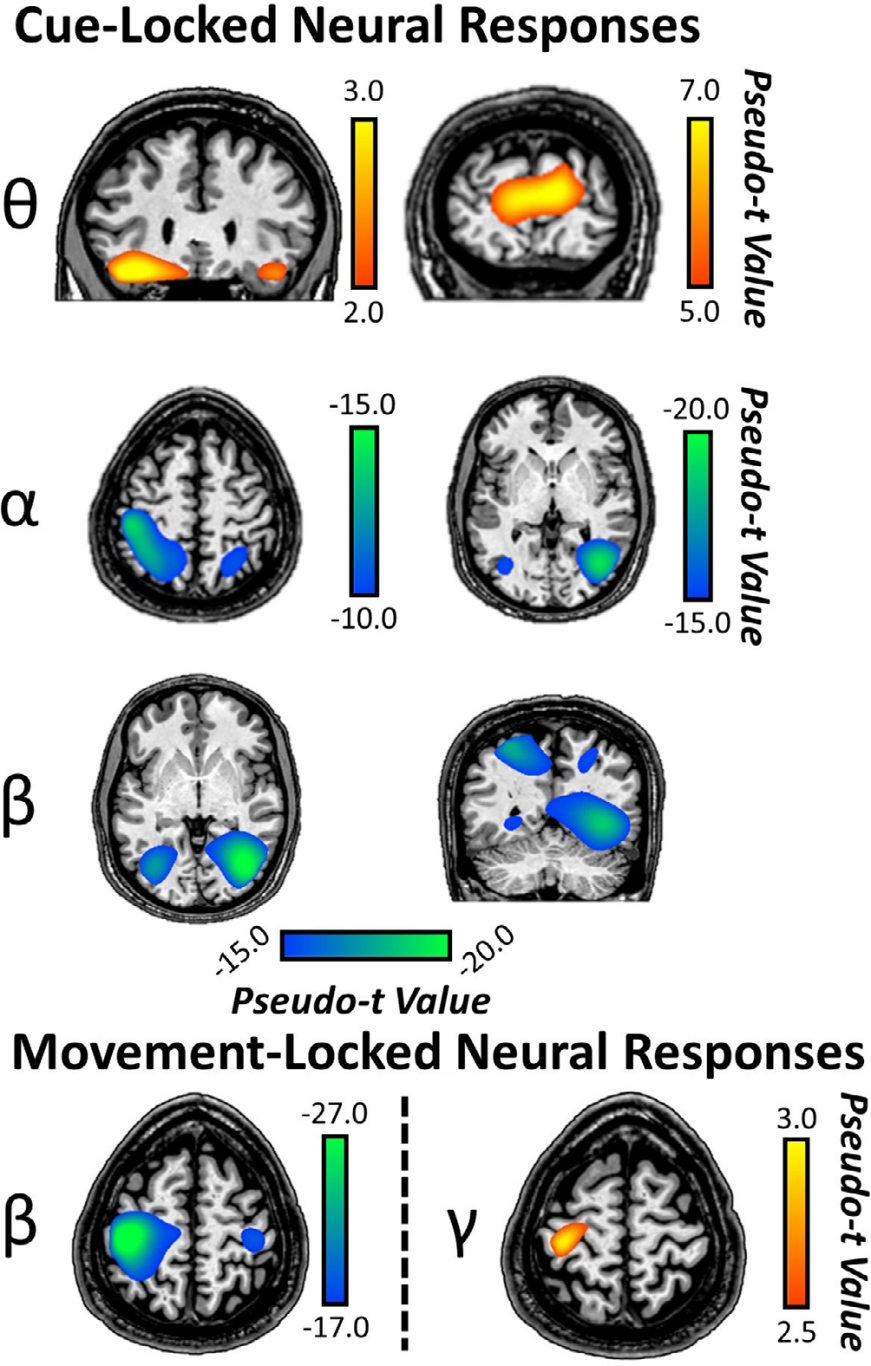
Cue‐ and movement‐locked oscillatory activity during target processing. (Top) Grand‐averaged beamformer images (pseudo‐t) for cue‐locked theta, alpha, and beta activity across both conditions and all participants revealed increases in theta activity in bilateral IFG and visual cortices. In contrast, decreases in alpha activity were observed in bilateral superior parietal lobules stretching anteriorly, and lateral occipital cortices. Decreases in beta activity were also observed in lateral occipital cortices and intraparietal sulci bilaterally. (Bottom) Grand‐averaged beamformer images (pseudo‐t) for the movement‐locked beta and gamma activity across both conditions and all participants revealed the well‐known perimovement beta ERD in bilateral precentral gyri (stronger contralateral to movement). Further, increases in gamma movement‐related synchrony (i.e., MRGS) were observed in the precentral gyrus contralateral to movement [Color figure can be viewed at http://wileyonlinelibrary.com]

To identify the brain regions generating the significant sensor‐level oscillations, these time frequency windows were imaged using a beamformer. The resulting maps were grand‐averaged across participants and these “condition‐invariant” responses are shown in Figure 3. In regard to cue‐locked responses, strong increases were observed across conditions in theta activity (4–8 Hz) from 425 to 675 ms in the bilateral inferior frontal gyri (IFG) and bilateral primary visual cortices. In contrast, strong decreases in alpha activity (8–14 Hz) from 300 to 900 ms were observed across conditions in the bilateral superior parietal lobules stretching anteriorly and the lateral occipital cortices (Figure 3). Finally, strong decreases in beta activity (300–900 ms) were observed in the bilateral intraparietal sulci and lateral occipital gyri (Figure 3).

As per the movement‐locked responses, we focused our source reconstruction on the temporal windows with the strongest perimovement beta ERD and MRGS responses. We did not image the PMBR as it was beyond the scope of the study since it occurs after movement. For the ERD, we imaged 16–26 Hz from −400 to 200 ms, which revealed strong ERD responses in the bilateral precentral gyri. As per the MRGS, we imaged a 66–76 Hz window from −75 to 75 ms and this showed that responses were centered on the left precentral gyrus (M1) (Figure 3).

### 3.4 Relationship between top‐down validity effects on behavior and neural oscillations

**Figure 4:**
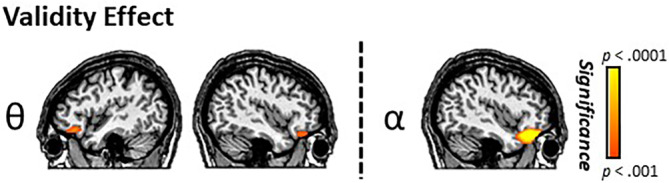
Conditional effects during target processing in the prefrontal cortex. The results from the whole‐brain analyses of condition‐related effects (paired *t* tests) on theta (left) and alpha (right) activity are shown. Significant conditional differences (i.e., validity effects) in bilateral inferior frontal gyri (IFG) theta activity were observed, with participants exhibiting stronger theta increases during invalid relative to valid trials (*p* < .001, corrected). Similarly, participants had significantly stronger alpha increases during invalidly relative to validly cued targets in the right IFG (*p* < .001, corrected) [Color figure can be viewed at http://wileyonlinelibrary.com]

Paired‐sample *t* tests were used to statistically evaluate validity effects (i.e., invalid vs. valid) for cue‐locked responses in the theta, alpha, and beta frequency bands using a whole‐brain approach. In the theta range (425–675 ms), this analysis revealed validity effects in the bilateral IFG, where theta increases were stronger during invalid relative to valid trials (*p* < .001, corrected; Figure 4). Alpha band validity effects were also observed in the right IFG, such that there were greater increases in alpha activity during invalid relative to valid trials (*p* < .001, corrected). There were no significant validity effects observed in the beta range. From these maps showing robust validity effects in frontal cortices (i.e., theta and alpha), the peak voxel in each respective IFG cluster was identified and we extracted its value for validly cued and invalidly cued trials separately. These data were then used to compute validity difference scores (i.e., invalid– valid) per response and participant.

For completeness, validity effects were also investigated for the perimovement beta ERD and MRGS responses from the movement‐locked analysis using the same method as described above for the cue‐locked data. These whole‐brain, paired‐sample *t* tests revealed no significant validity effect in any region.

**Figure 5:**
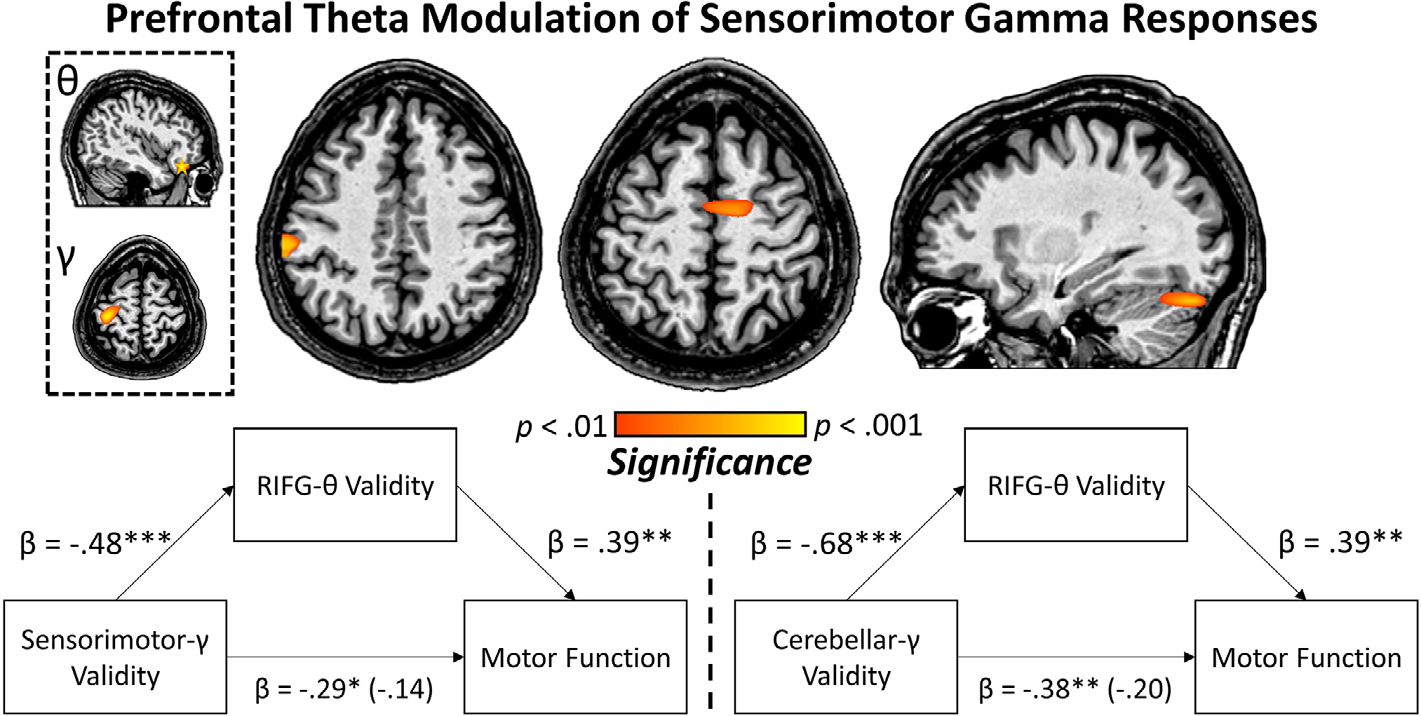
Relationship between prefrontal theta and movement‐related gamma. (Top) Whole‐brain correlation between the validity difference effect theta cluster in the right inferior frontal gyri (IFG; extracted peak is denoted by yellow star in the top image within the dashed box—from Figure 4) and whole‐brain gamma validity difference maps (image below the right IFG map in dashed box) revealed significant clusters in the sensorimotor cortex contralateral to movement, right supplementary motor area (SMA), and right cerebellum. This indicates that a greater theta validity difference effect in the right IFG is associated with an increased gamma validity difference effect in a distributed sensorimotor network. (Bottom) Group‐wise gamma validity in the contralateral sensorimotor cortex and right cerebellum, extracted from the peak voxel of the voxel‐wise correlation maps described above, was significantly associated with overall motor function measured independently using a neuropsychological testing battery. Mediation analyses using regression revealed that the theta validity difference effect in the right IFG fully mediated the sensorimotor gamma validity/motor function and cerebellar gamma validity/motor function relationships across participants. These analyses survived bootstrapping of 5,000 samples with confidence intervals of 95% [Color figure can be viewed at http://wileyonlinelibrary.com]

To ascertain the impact of additional top‐down modulation of motor circuits during invalid trials, the theta and alpha difference scores (invalid–valid) for the aforementioned cue‐locked IFG peaks were entered into voxel‐wise correlation analyses with the whole‐brain perimovement beta ERD and MRGS validity difference maps (i.e., invalid–valid) computed using the movement‐locked data. For theta, this revealed a robust relationship such that as validity difference effect scores increased in the right IFG, validity‐related effects on MRGS in the contralateral sensorimotor cortex, right supplementary motor area (SMA) and right cerebellum also tended to increase (*p* < .01, corrected; Figure 5). That is, the greater the increase in right IFG theta activity during the processing of invalidly relative to validly cued targets, the greater the increase in sensorimotor gamma activity during movement onset to invalidly relative to validly cued targets. Interestingly, this pattern held only for the right IFG theta validity response and MRGS activity; there were no significant correlations with the left IFG theta validity data, frontal alpha validity data, nor correlations with the whole‐brain perimovement beta ERD validity difference maps (i.e., invalid–valid) using the same statistical threshold (*p* < .01, corrected). Thus, the pattern was unique to right IFG theta (cue‐locked) and MRGS (movement‐locked) validity maps. Finally, to ensure the IFG responses were not related to ocular artifacts (e.g., eye movements), we conducted a supplementary analysis to identify any remaining ocular artifacts following the application of SSP. This analysis is reported in the supplementary materials and shows that eye movements were not detectable in the time‐domain averaged data following SSP (Figure S1).

### 3.5 Relationship to independent measures of motor function

Interestingly, the strength of MRGS extracted from sensorimotor regions in these whole‐brain statistical maps was also significantly related to overall motor functioning on independent neuropsychological assessments (i.e., RT on the Grooved Pegboard test with dominant and nondominant hands). Specifically, validity difference scores (i.e., invalid–valid) were computed for the MRGS peak voxels derived from the significant frontal theta/gamma validity correlation in the contralateral sensorimotor cortex, right SMA, and right cerebellum (Figure 5). Interestingly, there was no significant association among SMA validity in the gamma band and overall motor function (*r* = −.05, *p* = .685), but there was a strong relationship among overall motor function and validity in the right cerebellum (*r* = −.38, *p* = .003) and contralateral sensorimotor cortex (*r* = −.29, *p* = .025) such that stronger validity effects in these regions were associated with higher motor functioning. To empirically link these indices (i.e., prefrontal theta validity, MRGS validity in sensorimotor and cerebellar regions, and motor function), we next conducted a mediation analysis (Baron & Kenny, 1986). Briefly, a mediation analysis uses regression to test a causal model by which the mediator variable (i.e., prefrontal theta validity in this case) elicits an outcome (i.e., relationship between sensorimotor activity/motor function). We hypothesized a full mediation of the relationship among MRGS validity difference effects in contralateral sensorimotor cortex and right cerebellum on behavior through the mediator (i.e., prefrontal theta validity). Our results supported this and indicated a full mediation of MRGS validity in the contralateral sensorimotor cortex and right cerebellum on overall motor functioning that survived bootstrapping of 5,000 samples (95% CI: −.0334 to −.0028 and − .0368 to −.0002 for contralateral sensorimotor cortex and right cerebellum, respectively; Figure 5). Importantly, this suggests that increased theta validity difference effects in the right IFG drive the relationship with MRGS across a distributed motor network as well as independently measured motor functioning.

## 4 DISCUSSION

In the current study, we investigated the modulation of primary motor responses by higher order attention networks using an attentional reorienting paradigm. Using advanced oscillatory analyses, we observed robust cue‐locked and movement‐locked oscillatory responses in frontoparietal, occipital, and motor networks, respectively. Further, significant validity effects (i.e., invalid–valid) were observed in bilateral IFG in the theta band and in the right IFG in the alpha frequency range. Within these clusters, we calculated a validity difference effect score (i.e., invalid–valid), which quantifies the process of disengaging and shifting attention during unexpected stimuli presentation. Importantly, the theta validity difference score in the right IFG significantly predicted the validity difference effect of gamma activity in a distributed motor network, and further prefrontal theta activity fully mediated the relationships between overall motor functioning and MRGS in the contralateral sensorimotor and right cerebellar cortices. Below, we discuss the implications of these novel findings for understanding how frontal cognitive control processes influence bottom‐up motor responses.

Previous investigations of the MRGS response have shown that it is modulated by changes in basic movement parameters such as the force, frequency and pacing of the movement to be performed (Cheyne et al., 2008; Muthukumaraswamy, 2010). It has only recently been suggested to be manipulated by higher order parameters. Such studies have shown conditional modulations of the MRGS in times of response conflict compared to no conflict, in terms of changes in MRGS peak latency and/or peak frequency as a function of task demands (Gaetz et al., 2013; Grent‐'t‐Jong et al., 2013; Heinrichs‐Graham et al., 2018). However, the current study is the first to probe higher order modulation of the MRGS as a function of attentional reallocation. Importantly, we observed strong MRGS activity in contralateral M1, but surprisingly the different task demands (i.e., valid and invalid targets) did not significantly alter the MRGS response.

Our most important finding was likely the significant link between theta activity in the right IFG and gamma activity across a network of sensorimotor regions during attentional reorienting. Of note, the Posner cueing task (Posner, 1980) is known to effectively recruit nodes of the dorsal and ventral attention networks for the active engagement of visual resources, and the reorienting of those resources toward alternate locations. While engagement of frontoparietal networks was observed during target processing in the current study, our results implicated prefrontal areas specifically in the reorienting of attention to invalidly presented target stimuli. In other words, we observed conditionally specific increases in bilateral IFG, such that greater increases in frontal theta and alpha oscillatory activity were apparent during invalid relative to validly cued trials, but not in other regions.

Neuroimaging, neuropsychological, and lesion studies have broadly implicated the IFG in executive control. Specifically, right‐lateralized IFG responses have been linked to performance on response inhibition, task switching, and memory retrieval paradigms, with a goal of suppressing inappropriate responses (Aron, Robbins, & Poldrack, 2004, 2014; Hampshire, Chamberlain, Monti, Duncan, & Owen, 2010; Proskovec, Wiesman, & Wilson, 2019). Additionally, the processing of cues and shifting of attention to trigger task‐relevant behavior has long been associated with increases in the ventral attention network as evidenced by fMRI (Corbetta et al., 2008; Corbetta & Shulman, 2002; Hampshire et al., 2010; Petersen & Posner, 2012) and more recently, MEG (Proskovec et al., 2018). Evidence has also suggested that recruitment of the prefrontal cortices reflects a hierarchical modulation of posterior brain regions by prefrontal activity (Brass, Ullsperger, Knoesche, von Cramon, & Phillips, 2005; Koechlin, Ody, & Kouneiher, 2003; Miller, 2000; Miller & D'Esposito, 2005; Pessoa, Kastner, & Ungerleider, 2003). For example, Brass et al. measured both fMRI and event‐related potentials (ERPs) during a modified task‐switching paradigm, and found that prefrontal dipoles in the right IFG and left inferior frontal junction largely contributed to the ERP effect earlier than a parietal dipole (Brass et al., 2005).

Our oscillatory analysis of the subprocesses of attentional reallocation in the current study implicated theta band activity in the disengagement of attention from invalidly cued locations. We observed robust recruitment of theta band activity in the bilateral IFG during target processing and further, larger validity difference effects in right frontal theta responses were significantly related to increases in the MRGS validity difference effect across a distributed motor network, including the contralateral sensorimotor cortices, right SMA, and right cerebellum. Previous studies have suggested that functional coupling of theta and gamma oscillations may be critical to cognitive processing and congruency effects in particular (Bramson, Jensen, Toni, & Roelofs, 2018; Friese et al., 2013; Lisman & Jensen, 2013; Tort, Komorowski, Manns, Kopell, & Eichenbaum, 2009). For instance, invasive neurophysiological studies in animals have revealed that the relationship between theta phase and gamma amplitude (i.e., cross‐frequency coupling) increases as a function of learning and is associated with food reward locations (Lisman & Jensen, 2013; Tort et al., 2009). Long‐term memory has also been associated with increases in frontal theta and posterior gamma coupling in humans (Friese et al., 2013; Lisman & Jensen, 2013). While cross‐frequency coupling was not directly investigated in the current study, the mediation relationship with M1 gamma/motor functioning suggests its potential role beyond just attentional reorienting.

In conclusion, the current study sought to investigate the potential for a higher order attentional influence on movement‐related oscillatory responses. To date, few studies have probed the influence of higher order cognitive processes on movement‐related gamma oscillations. In that regard, we found that a metric of attentional reorienting in the right, but not left IFG was significantly related to gamma activity in a sensorimotor network during movement. Importantly, as frontal theta validity increased, the gamma validity difference effect in the contralateral sensorimotor cortex, right SMA, and right cerebellum also increased. In addition, the power of gamma activity observed in the contralateral sensorimotor and right cerebellar cortices was significantly related to overall motor functioning and this relationship was driven by increases in prefrontal theta validity. Taken together, these findings provide new and important evidence for frontal theta and posterior gamma interactions during attentional reorienting. Prefrontal involvement during the reorienting of attention may be crucial for rapid and adaptive control of motor execution.

## CONFLICT OF INTEREST

The authors report no financial, institutional, or commercial conflicts of interest.

## Supporting information


**Figure S1** Time domain averaged data for participants showing strong theta responses in the inferior frontal gyri (IFG). To ensure the IFG responses were not related to saccadic eye movement artifacts, we time domain averaged the artifact‐corrected data separately by condition (valid and invalid) and side that the target was presented on (left and right). Briefly, prior to averaging, we removed eye movement artifacts (e.g., blinks and saccadic eye movements) using signal‐space projection (SSP). SSP was also used to remove the cardiac artifact. We then rejected trials containing high amplitude and/or gradient values (see Methods) and time domain averaged the remaining trials per condition and side of target. Shown above are the averages; for each of these four participants, we show the data for each gradiometer across the entire array, as well enlarged views of two frontal gradiometers that typically show the largest ocular artifacts. In each plot, time is shown on the x‐axis in ms and amplitude is shown on the y‐axes in femtoTesla (fT), all data has been filtered using a 20 Hz low‐pass to focus on the spectral range where eye artifacts are the most influential. Gray boxes denote the temporal window used for imaging prefrontal theta activity (i.e., 425–675 ms). For the two enlarged sensors, we present data separately for target left and target right conditions, and in each have overlaid the valid (no saccade) and invalid (saccade) trials. If a significant ocular artifact remained in the data, it should be present only in the invalid condition and thus should be easily apparent in the overlaid waveforms. In conclusion, these data show that our SSP approach was adequate for removing saccadic and other artifacts from the data, and support the conclusion that our theta‐frequency IFG responses were not likely affected by such artifacts. Finally, note that we plotted the left and right target location data separately, as the polarity of the artifact differs based on the direction of the saccade, and thus averaging them together could eliminate the artifact if one were present.

## Data Availability

The data that support the findings of this study are available from the corresponding author upon reasonable request.
