# Supplementary material for: Prefrontal theta modulates sensorimotor gamma networks during the reorienting of attention
Source: Hum Brain Mapp. 2019 Oct 17;41(2):520–9. doi: 10.1002/hbm.24819 (PMC7268018; doi:10.1002/hbm.24819)
Supplement: Supplementary file 1 — Figure S1 Time domain averaged data for participants showing strong theta responses in the inferior frontal gyri (IFG). To ensure the IFG responses were not related to saccadic eye movement artifacts, we time domain averaged the artifact‐corrected data separately by condition (valid and invalid) and side that the target was presented on (left and right). Briefly, prior to averaging, we removed eye movement artifacts (e.g., blinks and saccadic eye movements) using signal‐space projection (SSP). SSP was also used to remove the cardiac artifact. We then rejected trials containing high amplitude and/or gradient values (see Methods) and time domain averaged the remaining trials per condition and side of target. Shown above are the averages; for each of these four participants, we show the data for each gradiometer across the entire array, as well enlarged views of two frontal gradiometers that typically show the largest ocular artifacts. In each plot, time is shown on the x‐axis in ms and amplitude is shown on the y‐axes in femtoTesla (fT), all data has been filtered using a 20 Hz low‐pass to focus on the spectral range where eye artifacts are the most influential. Gray boxes denote the temporal window used for imaging prefrontal theta activity (i.e., 425–675 ms). For the two enlarged sensors, we present data separately for target left and target right conditions, and in each have overlaid the valid (no saccade) and invalid (saccade) trials. If a significant ocular artifact remained in the data, it should be present only in the invalid condition and thus should be easily apparent in the overlaid waveforms. In conclusion, these data show that our SSP approach was adequate for removing saccadic and other artifacts from the data, and support the conclusion that our theta‐frequency IFG responses were not likely affected by such artifacts. Finally, note that we plotted the left and right target location data separately, as the polarity of the artifact differs based on the dir [file HBM-41-520-s001.pdf]

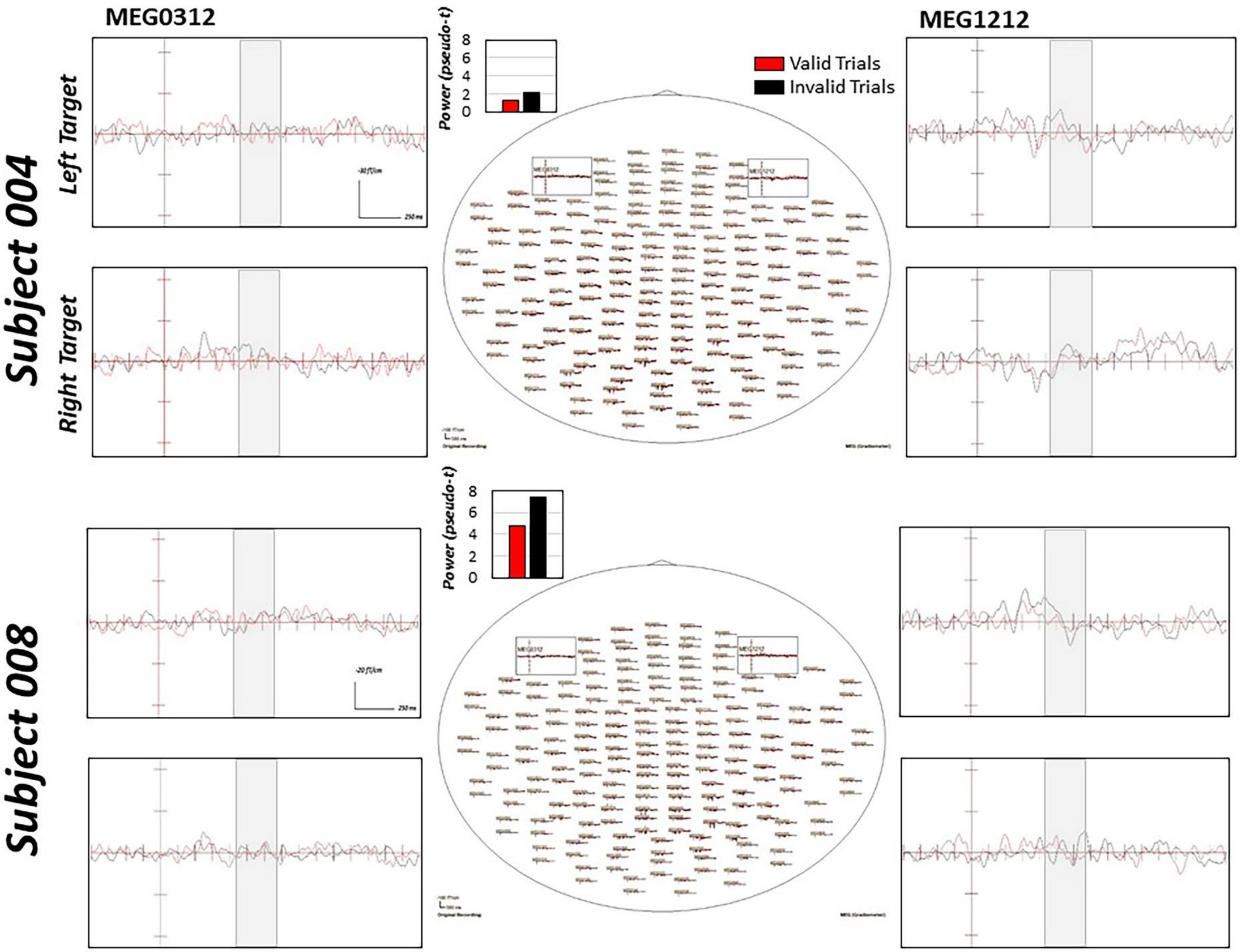
MEG0312
MEG1212
Valid Trials
Invalid Trials
Power (pseudo-t)
8
6
4
2
0
Subject 004
Left Target
Right Target
Subject 008
250 ms
MEG0312
MEG1212

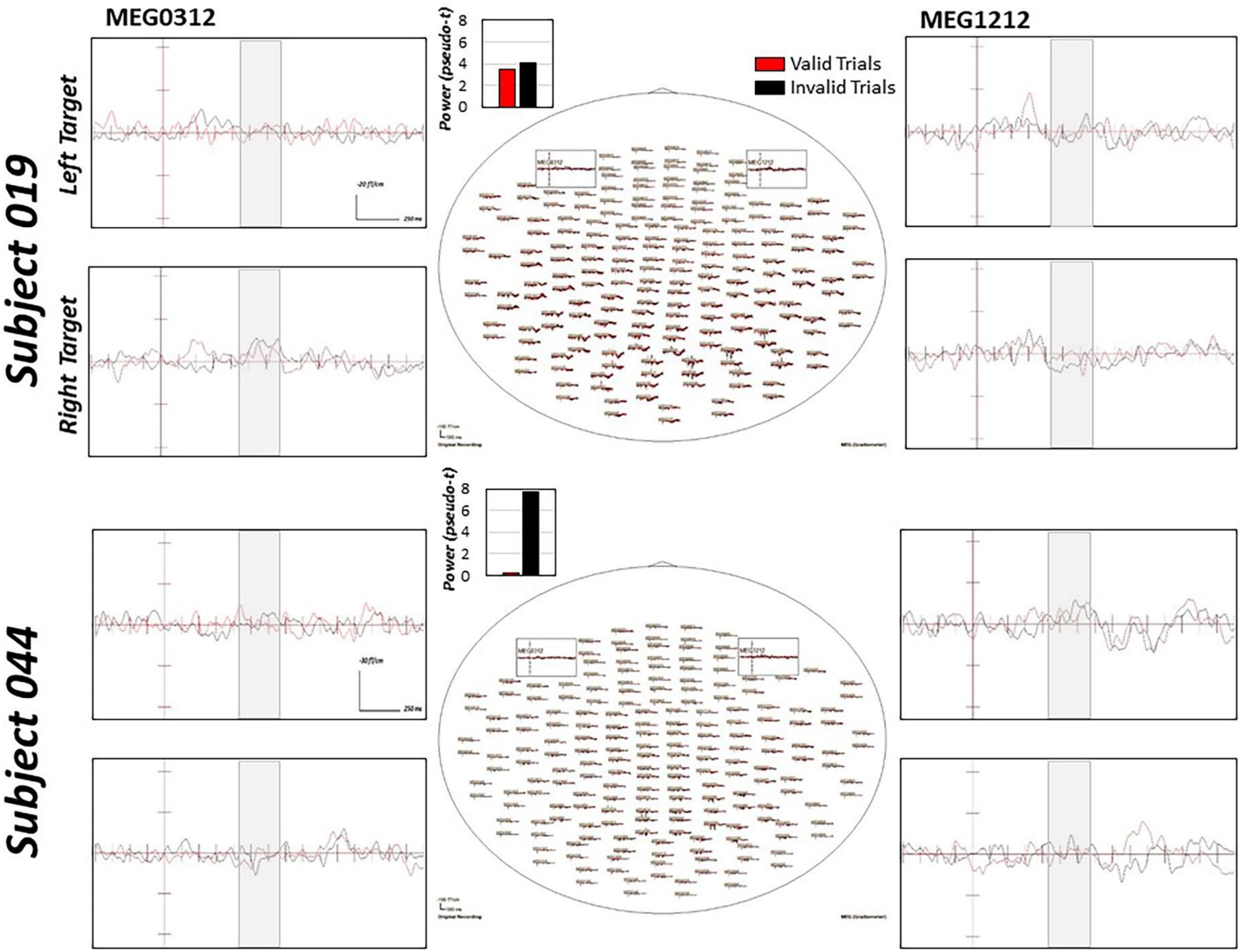

MEG0312
MEG1212
Subject 019
Left Target
Right Target
Power (pseudo-t)
8
6
4
2
0
Valid Trials
Invalid Trials
Subject 044
Power (pseudo-t)
8
6
4
2
0

**Figure S1:** Time domain averaged data for participants showing strong theta responses in the inferior frontal gyri (IFG). To ensure the IFG responses were not related to the saccadic eye movement artifact, we time domain averaged the artifact-corrected data separately by condition (valid and invalid) and side that the target was presented on (left and right). Briefly, prior to averaging, we removed eye movement artifacts (e.g., blinks and saccadic eye movements) using signal-space projection (SSP). SSP was also used to remove the cardiac artifact. We then rejected trials containing high amplitude of gradient values (see Methods) and time domain averaged the remaining trials per condition and side of target. Shown above are the averages; for each of these four participants, we show the data for each gradiometer across the entire array, as well enlarged views of two frontal gradiometers that typically show the largest ocular artifacts. In each plot, time is shown on the x-axis in ms and amplitude is shown on the y-axes in femtoTesla (fT), all data has been filtered using a 20 Hz low-pass to focus on the spectral range where eye artifacts are the most influential. Gray boxes denote the temporal window used for imaging prefrontal theta activity (i.e., 425-675 ms). For the two enlarged sensors, we present data separately for target left and target right conditions, and in each have overlaid the valid (no saccade) and invalid (saccade) trials. If a significant ocular artifact remained in the data, it should be present only in the invalid condition and thus should be easily apparent in the overlaid waveforms. In conclusion, these data show that our SSP approach was adequate for removing saccadic and other artifacts from the data, and support the conclusion that our theta-frequency IFG responses were not likely affected by such artifacts. Finally, note that we plotted the left and right target location data separately, as the polarity of the artifact differs based on the direction of the saccade, and thus averaging them together could eliminate the artifact if one were present.
